# Motivations Associated with Food Choices and Eating Practices

**DOI:** 10.3390/foods10040834

**Published:** 2021-04-12

**Authors:** Raquel P. F. Guiné

**Affiliations:** CERNAS Research Centre, Department of Food Industry, Polytechnic Institute of Viseu, 3504-510 Viseu, Portugal; raquelguine@esav.ipv.pt

The principal reason that influences people’s eating characteristics is to satisfy basic body stimuli, like feeling hunger and the need for satiety. Nevertheless, people’s food choices are not determined solely by physiological needs or nutritional demands. Actually, in addition to the central factors that lay behind the act of eating, it is possible to enumerate an extensive diversity of other factors which also condition the food choices. The human behaviors regarding foods are associated with a number of reasons, some of sociological nature and others of psychological essence. Hence, it is important to better understand the different psychic and social motivations that determine people’s eating patterns, either in relation to their food choices or to their eating habits. The possible different types of motivations that shape food consumption can include areas such as, but not exclusively, health motivations, economic factors, emotional aspects, cultural influences, marketing and commercials or environmental concerns.

Presently, the close relation between eating habits and the prevention of a large number of pathologies is well-established. Unhealthy diets comprise minimal fruit and vegetable intake and excessive consumption of processed convenience foods high in salt, fat and sugar. However, an excess of fats, sugars or salt has been associated with many chronic diseases [1,2]. It has been demonstrated that eating inappropriate amounts of fats, and particularly saturated and trans fats, constitutes an increased risk of heart diseases such as stroke, atherosclerotic vascular diseases and, in particular, coronary heart disease, cardiac dysfunction (indirect and direct cardiac effects, including inflammation, hypertrophy, fibrosis and contractile dysfunction) or increased LDL (Low Density Lipoproteins) cholesterol and triglycerides. Salt has also been recognized as a major contributor to cardiovascular diseases, as it progressively raises blood pressure levels with age. On the other hand, the presence of added sugars in the diet is associated with an increased risk of obesity and other obesity-related chronic diseases, including type 2 diabetes. Additionally, an excess of weight has been identified as a major risk factor for a range of preventable chronic diseases, including cardiovascular disease, cancer, osteoarthritis or diabetes. On the other hand, the insufficient ingestion of fruits and vegetables, as a result of modern trends towards the consumption of high levels of processed convenience foods, results in deficiencies in vitamins, dietary minerals, dietary fibers and bioactive substances such as, for example, antioxidants, among other extremely important food constituents.

It is unquestionable that food is essential to provide the human body with the energy it needs to function, as well as macro and micro components and bioactive components with major roles in maintaining health. However, food is also recognized as an inseparable part of traditions and culture, as well as social environments, and hence, eating has a strong emotional component. Some people may have had certain eating habits for so long that they do not even realize they are unhealthy. On the other hand, for many people, even if a need to change their eating habits is identified, it might be very hard to do it for a number of reasons: the present habits have become part of their daily life, so they do not think much about them; they want to change but familiar or friends’ influences may overcome their intentions; the role of publicity and marketing must also be accounted for; or simply they have become addicted to bad foods and it is difficult to overcome this addiction.

One’s emotional status influences all features of human life, and naturally also eating behavior. People tend to establish regular dietary patterns or make different food choices adaptable to their emotional status or temporary moods. Emotional eating is associated with a trend to overeat as a compensation for negative emotions, like, for example, depression, anxiety or irritability. Conversely, for other individuals, feelings of sadness, loneliness or depression can block their appetite, preventing them from ingesting appropriate amounts of the nutrients essential for the correct body functioning. Therefore, emotions assume an incredibly relevant role in people’s eating behavior [3,4].

Economic factors have also been demonstrated to have a modulatory effect on eating patterns, evidencing differences, for example, at the level of low household income families when compared to those societies with a higher average income and characterized by a higher level of industrialization [5]. However, other factors, besides price, also contribute to define consumer trends, such as availability or cultural aspects. In the present global markets, food products are commercialized amongst many different countries and across cultures, and the role of tradition is also relevant, especially when it comes to protected regional foods. The willingness to consume traditional food products is directly related to the concept of authenticity and how this is perceived by consumers [6,7,8].

The sustainability across the food supply chain is also driving modern consumers towards sustainable or green consumer behavior, which relates to food choices that aim at preserving the environment and ecosystems’ biodiversity. Sustainable consumers refuse to buy and consume foods that bring detrimental effects to the environment, and instead look for products which cause a minimal impact on the planet, or even that are beneficial for global sustainability. Hence, nowadays a great deal of importance is given to the development of nutritionally improved foods, with good acceptability and which at the same time bear additional environmental impacts. This is the case with the utilization of food industry by-products, that otherwise would have to be discarded, being incorporated into new foods [9].
